# Evaluation of *Cinnamomum osmophloeum* Kanehira Extracts on Tyrosinase Suppressor, Wound Repair Promoter, and Antioxidant

**DOI:** 10.1155/2015/303415

**Published:** 2015-03-09

**Authors:** Man-Gang Lee, Su-Yu Kuo, Shih-Yu Yen, Hsia-Fen Hsu, Chung-Hang Leung, Dik-Lung Ma, Zhi-Hong Wen, Hui-Min David Wang

**Affiliations:** ^1^Department of Marine Biotechnology and Resources, National Sun Yat-sen University, Kaohsiung 804, Taiwan; ^2^Division of Urology, Department of Surgery, Zuoying Branch of Kaohsiung Armed Forces General Hospital, Kaohsiung 813, Taiwan; ^3^Department of Fragrance and Cosmetic Science, Graduate Institute of Natural Products, Center for Stem Cell Research, Kaohsiung Medical University, No. 100, Shih-Chuan 1st Road, San-Ming District, Kaohsiung 80708, Taiwan; ^4^Department of Bioscience Technology, Chang Jung Christian University, Tainan 711, Taiwan; ^5^Metal Industries Research & Development Centre, Kaohsiung 812, Taiwan; ^6^State Key Laboratory of Quality Research in Chinese Medicine, Institute of Chinese Medical Sciences, University of Macau, Macau; ^7^Department of Chemistry, Hong Kong Baptist University, Kowloon Tong, Hong Kong; ^8^Marine Biomedical Laboratory & Center for Translational Biopharmaceuticals, Department of Marine Biotechnology and Resources, National Sun Yat-sen University, 70 Lien-Hai Road, Kaohsiung 804, Taiwan

## Abstract

*Cinnamomum osmophloeum* Kanehira belongs to the Lauraceae family of Taiwan's endemic plants. In this study, *C. osmophloeum* Kanehira extract has shown inhibition of tyrosinase activity on B16-F10 cellular system first. Whether extracts inhibited mushroom tyrosinase activity was tested, and a considerable inhibition of mushroom tyrosinase activity by *in vitro* assays was presented. Animal experiments of *C. osmophloeum* Kanehira were carried out by observing animal wound repair, and the extracts had greater wound healing power than the vehicle control group (petroleum jelly with 8% DMSO, w/v). In addition, the antioxidant capacity of *C. osmophloeum* Kanehira extracts *in vitro* was evaluated. We measured *C. osmophloeum* Kanehira extract's free radical scavenging capability, metal chelating, and reduction power, such as biochemical activity analysis. The results showed that a high concentration of *C. osmophloeum* Kanehira extract had a significant scavenging capability of free radical, a minor effect of chelating ability, and moderate reducing power. Further exploration of the possible physiological mechanisms and the ingredient components of skincare product for skin-whitening, wound repair, or antioxidative agents are to be done.

## 1. Introduction

Skin, made up of three layer cells including, epidermis, dermis, and hypodermis, is the largest vertebrates organ in the human body. Human skin is commonly exposed to oxidative stresses from solar ultraviolet (UV) radiation and free radicals as well as its induced cellular reactive oxygen species (ROS) [1, 2], which are the common reasons for tumor genesis or skin aging. To protect skin from UV radiation, skin operates complex defense system including skin thickening, pigment synthesis, and a network of nonenzymatic and enzymatic antioxidative mechanisms [2]. In addition to a significant responsibility, in the prevention of human skin UV-caused damage, which increases melanocytes transfer of melanosomes to keratinocytes, melanin determines skin color [3]. Hyperpigmentation is commonly cared with therapeutic drugs or cosmetics of pigment-reducing or skin-whiten abilities. During the melanin synthesis processes, tyrosinase is classified to be the rate-limiting oxidase at first two steps [4]. It catalyzes the pigments production such as eumelanin and phenomelanin. Two types of pigments production were reported, including the L-tyrosine hydroxylation to 3,4-dihydroxy-L-phenylalanine (L-DOPA) and then the L-DOPA oxidation to dopaquinone (a biochemical precursor to pigments) [5]. In the active site for tyrosinase, two copper ions are essential to catalyze colorful pigments or melanin by oxidative stress. To antagonize tyrosinase activity can reduce the syndrome of hyperpigmentation and dermatological disorders.

Skin as the first immune defense line of human plays a noteworthy role in avoiding various biological, chemical, mechanical, and physical damages [1, 2]. Chronic or acute severe injuries on the skin, such as abrasions, burns, leg ulcers, or lesions, in consequence considerable losses of dermal tissues pose huge challenges to the therapeutic processes. Keratinocytes in epidermis and fibroblasts in dermis are the first stop for body protection against external stimulus or for the skin wound healing [6]. In terms of wound healing, wound closure is known to be initiated by fibroblast migration from its margins. Based on the migratory force, resistance from the regenerated tissue may lead to fibroblast differentiation [7], which is featured by the local expression profiles of skin cells, such as several growth factors and the extracellular matrix. Skin wound healing is a cutting edge study for many medicine fields [8].

Smoking factors, salted food, or environmental toxicants bring about various oxidative stresses to human being [9]. The level of excessive free radicals produces a high oxidative stress which is a negative effect against the normal skin and results in aging or some diseases. Through biochemical processes, the intracellular physiological oxidants are engendered from nonenzymatic systems such as those involving enzymatic catalysis, transition metals, various oxidases that transformed them into the reactive nitrogen species, or reactive oxygen species [1]. If antioxidants are invigorated, they can significantly prevent or reduce the oxidative pressure damages [10]. There are several important components constructed to cellular membrane lipids from the phospholipids, membrane proteins, polyunsaturated fatty acids, cholesterol, and nucleic acids [11]. Excessive free radicals and ROS cause oxidative pressure injury on lipids, proteins, and DNA, and the damage eventually induced cellular damage, aging, neural disorders, diabetes, atherosclerosis, inflammatory, cancer, and cardiovascular disease, especially unwanted pigment accumulation [12].

**Figure 1:**
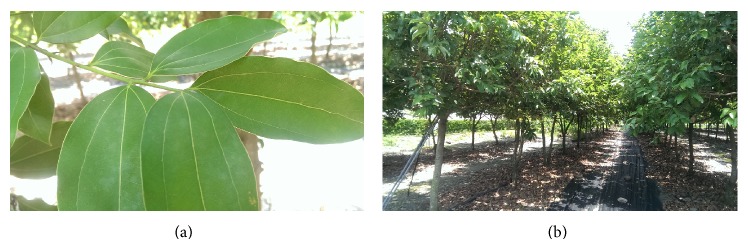
The photos of* Cinnamomum osmophloeum* Kanehira. (a) Leaves. (b) A whole plant.

*Cinnamomum osmophloeum* Kanehira is commonly recognized as indigenous cinnamon or pseudocinnamomum. The natural plant is native to broad-leaved forests of Taiwan's endemic plants (Figure 1) and lots of exercises as a Chinese herbal medicine, including tannin, resin, mucilage, sugar, and essential oil, among which essential oils had excellent inhibitions on bacterial pharmacological characteristics [13]. This plant contains many nutrients such as manganese, dietary fiber, iron, and calcium, commonly used as a spice and flavoring agent for many foods [14]. Various biofunctional applications have been found that* C. osmophloeum *Kanehira has high anti-inflammatory and antioxidative properties which plays a key role in tissue repair of traditional medicine [15]. Moreover,* C. osmophloeum *Kanehira could be used as a xanthine oxidase inhibitor for management of hyperuricemia and related medicinal situations including gout, hence a potential drug [16].

## 2. Materials and Methods

### 2.1. Reagents and Materials

All the reagents were purchased from Sigma Chemical (St. Louis, MO), including dimethyl sulfoxide (DMSO), 1,1-diphenyl-2-picrylhydrazyl (DPPH), 3-(4,5-dimetylthiazol-2-yl)-2,5-diphenyl, tetrazolium bromide (MTT), 3-*tert*-butyl-4-hydroxyanisole (BHA), ethylenediaminetetraacetic acid (EDTA), FeCl_3_, FeCl_2_·4H_2_O, kojic acid, L-tyrosine, mushroom tyrosinase, potassium ferricyanide [K_3_Fe(CN)_6_], trichloroacetic acid and vitamin C, and other highest purity chemical buffers and reagents. Cell culture reagents were purchased from GIBCO BRL (Gaithersburg, MD), including fetal bovine serum (FBS) and Dulbecco's modified Eagle medium (DMEM).

### 2.2. Extraction of* C. osmophloeum* Kanehira Leaves

The plant specimen was authenticated by Ladies Biotech Co., LTD, where voucher specimens were kept. Dry leaves of* C. osmophloeum *Kanehira (0.6 kg) were sliced and soaked in 3 L ethyl alcohol for one day before further three ethyl alcohol extractions. After filtration, the extracts were evaporated to final weight of 8.49 g.

### 2.3. B16-F10 Melanoma Cell Cultures

The melanoma B16-F10 cells (BCRC 60031 in ATCC) were maintained at 37°C under 5% CO_2_ atmosphere by feeding the medium (10 mM HEPES, 13.4 mg/mL DMEM, 100 *μ*g/mL streptomycin sulfate, 143 U/mL benzylpenicillin potassium, and 24 mM NaHCO_3_, pH 7.1) with 10% FBS [17].

### 2.4. B16-F10 Cell Viability

MTT assay was used to evaluate the effects of cell viabilities for the treatments of* C. osmophloeum *Kanehira extracts [17]. Briefly, cells (6 × 10^3^ cells/well) were plated in 96-well plates for overnight. Cells were treated with either vehicle (DMSO) or indicated concentrations of eachsample for 24 h. Subsequently, 0.5 mg/mL MTT in 100 *μ*L of fresh medium was used to replace the medium and the reaction was performed in a 37°C cell culture incubator for 2 h. The generating crystals were dissolved in 100 *μ*L of DMSO with smooth shaking for 10 min in darkness. Finally, the absorbance (*A*) value of this reaction was detected at 595 nm by multiplate reader (UV-vis, BioTek, Winooski, VT). Cell viability (%) was formulated as follows:

(1) $$\begin{array}{c} \text{Cell}\;\text{viability}\left(\%\right)=100\times \frac{{\text{OD}}_{\text{sample}}}{{\text{OD}}_{\text{control}}}. \end{array}$$

### 2.5. B16-F10 Cellular Tyrosinase Activity

The tyrosinase activity was dependent on the dopachrome formation rate as previously described [17]. Melanoma B16-F10 cells (10^5^ cells/well) were seeded in a 12-well plate with 1,000 *μ*L of medium, and they were treated with indicated concentrations of extracts for 48 h. After PBS washing, B16-F10 cells lysed with 1% triton X-100/PBS and 50 *μ*L of 2 mM L-tyrosine were added. Standing for 3 h at 37°C in darkness, its absorbance at 490 nm was examined spectrophotometrically, where the tyrosinase activity evaluation formula was similar to 2.

### 2.6. B16-F10 Cellular Melanin Contents

The cellular melanin contents were measured with minor modifications as previously described [17]. Briefly, B16-F10 melanoma cells (2.5 × 10^5^ cells/well/1500 *μ*L of medium) plated in 6-well plates were treated with extracts and incubated for 48 h. After dissolving in 10% DMSO with 50 *μ*L of 2.0 N NaOH for 1 h at 90°C, cell lysates were centrifuged at 10,000 ×g for 10 min to collect the supernatants for melanin determination using spectrophotometer at 475 nm with similar formula to 2.

### 2.7. Mushroom Tyrosinase Activity

The mushroom tyrosinase activity was measured with minor modifications as previously described [18, 19]. The various concentrations of extracts were added: 2 *μ*L with 68 *μ*L of 50 mM phosphate buffer (pH 6.8), 10 *μ*L of 0.5 units/mL of mushroom tyrosinase, and 10 *μ*L of the mixture. The absorbance of the mushroom tyrosinase inhibition assay at 490 nm was determined at 5 min per interval until 30 minutes with a 96-well plate spectrophotometer, where kojic acid was regarded as a positive control. Mushroom tyrosinase activity (%) was formulated as follows:

(2) Mushroom  tyrosinase  activity(%) =100−100×A−B−C−DA−B,

where *A* is the OD value under no sample; *B* is the OD value under no sample and tyrosinase; *C* is the OD value under sample; and *D* is the OD value under sample but no tyrosinase.

### 2.8. Animal Experiments

Six-week-old male Wistar rats are used. These rats are kept on standard rat chow and water ad libitum for 1 week before challenging. The animal studies were performed under authorization from the Animal Use Committee of Kaohsiung Medical University. The experimental rats were housed on a 12/12-hour light-dark cycle with the air conditioner and adequate supply of food and water. Twelve rats were grouped into two sets, that is, petroleum jelly and* C. osmophloeum* Kanehira extracts (experimental groups). The wound healing of skin was measured with minor modifications as previously described [20]. After rats were anesthetized, its dorsal hair was shaved and the wounds of 1 cm in diameter were generated. After a back skin excising, the wounds of all experimental rats were quickly covered with the petroleum jelly (8% DMSO, w/v) or 0.8 mg extracts, where the petroleum jelly was used as a reference.

### 2.9. Measurement of the Wound Area

Digital camera (Coolpix P6000, Nikon, Japan) was used to record the progression of skin wound after 0, 1, 3, and 5 days with protocol parameters (aperture: F/7.2, shutter speed: 1/60). The wound dressing was not removed under healing period unless the substance was easy to detach manually. The area of each skin wound was determined by SPOT software (Diagnostic Instruments, Inc., Sterling Heights, MI, USA). Some random Sani-Chips were visualized on wound sites; however, wound size measurements were not interfered. The wound healing index was formulated as follows:

(3) $$\begin{array}{c} \frac{\text{Wound}\;\text{area}\;\text{of}\;\text{day}\;N}{\text{Wound}\;\text{area}\;\text{of}\;\text{day}\;0}\times 100\%. \end{array}$$

### 2.10. Determination Antioxidation Ability by DPPH^∙^ Radical Scavenging

The principle of antioxidation determination is based on the color change of DPPH to light yellow if free radicals are scavenged [21]. The more the light color rendered, the higher the antioxidant capacity from the component. Suitable concentration doses of* C. osmophloeum *Kanehira extracts were added to 1 *μ*L and with 99 *μ*L of DPPH solution. When DPPH reacted with antioxidants or vitamin C (positive control), it changed to reduced form and led to a lower absorbance at 517 nm. The scavenging activity (%) of DPPH radical was formulated as follows:

(4) $$\begin{array}{c} \text{Scavenging}\;\text{activity}\left(\%\right)=100\times \frac{\left(\text{O}{\text{D}}_{\text{control}}-\text{O}{\text{D}}_{\text{sample}}\right)}{\text{O}{\text{D}}_{\text{control}}}. \end{array}$$

### 2.11. Metal Chelating Activity

Metal chelating activity was measured with slight modifications as previously described [5]. Briefly,* C. osmophloeum* Kanehira extracts dissolved in DMSO were mixed with a reagent containing 10 *μ*L of 2 mM FeCl_2_·4H_2_O. To initiate the reaction with the addition of 20 *μ*L of 5 mM ferrozine, the solution was vigorously shaken and then it was stood for 10 min at room temperature. EDTA was regarded to be a positive control. Its absorbance at 562 nm was calculated as the chelating activity (%) and its method was alike to 4.

### 2.12. Reducing Power

The reducing power of* C. osmophloeum* Kanehira extracts was determined according to the previous method [21]. The extracts were incubated with 85 *μ*L of 67 mM phosphate buffer (pH 6.8) and 2.5 *μ*L of 20% K_3_Fe(CN)_6_ at 50°C for 20 min. After the addition of 160 *μ*L of trichloroacetic acid (10%), it was centrifuged at 3,000 ×g for 10 min to collect the supernatant (75 *μ*L) for reacting with 2% FeCl_3_ (25 *μ*L). BHA was regarded to be a positive control. Finally, its absorbance at 700 nm was measured by spectrophotometer.

### 2.13. Statistics

Data are indicated as a mean and standard deviation in triplicate at least. Its difference significance was evaluated by Student's *t*-test.

## 3. Results and Discussion

### 3.1. B16-F10 Cytotoxicity of* C. osmophloeum* Kanehira Extracts

**Figure 2:**
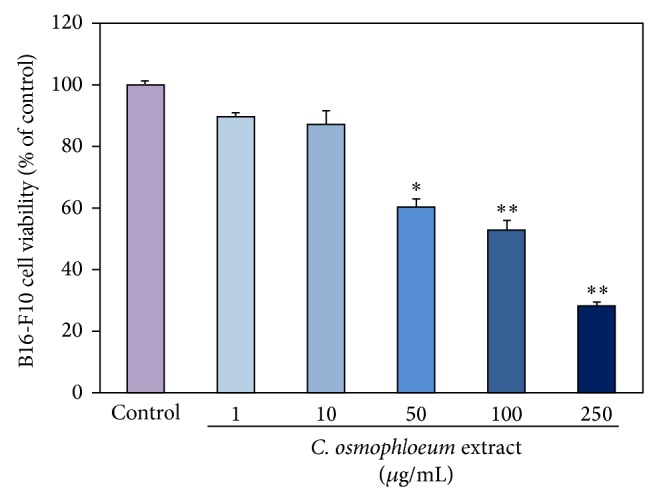
The cytotoxicity of* C. osmophloeum* Kanehira extracts on marine melanoma B16-F10 cells. Data: the mean value ± SD (triplicate values for three independent experiments); ∗ < 0.01, ∗∗ < 0.001.

Melanoma B16-F10 cells were cultured in the indicated doses of tested extracts (10, 25, 50, 100, and 200 *μ*g/mL). In Figure 2, the cell viability was determined by MTT assay. The proliferation of B16-F10 cell was inhibited by extracts in a dose-responsive manner ranging from 10 to 200 *μ*g/mL. When the mouse melanoma cells were incubated in a higher assay surrounding 100 *μ*g/mL, the viabilities of extract-treated B16-F10 cells were more than 50% at 48 h treatment, suggesting that extracts had discernable cytotoxic effect on mouse melanoma cells.

### 3.2. *C. osmophloeum* Kanehira Extracts on B16-F10 Cellular Tyrosinase Activity and Melanin Content

**Figure 3:**
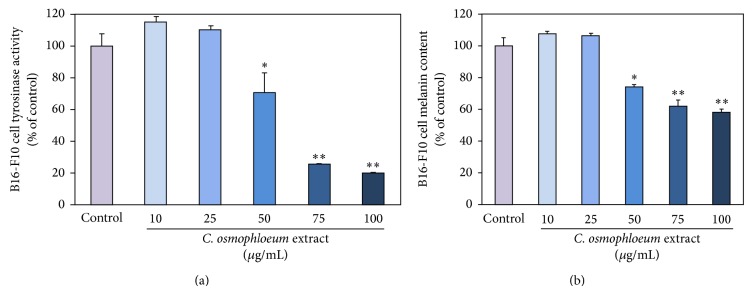
The inhibitory effects of various concentrations of* C. osmophloeum* extracts on B16-F10 cells. (a) The tyrosinase activity and (b) the melanin content of B16-F10 cells were incubated with indicated concentrations of* C. osmophloeum* extracts. Data: the mean value ± SD (triplicate values for three independent experiments); ∗ < 0.01, ∗∗ < 0.001.

We further investigated the* in situ* cellular tyrosinase and melanin suppressions of extracts. The melanin generation mechanisms contain the L-tyrosine hydroxylation and the L-DOPA oxidation to its corresponding dopaquinone to form pigment by additional multiple biosynthesis steps through the enzymatic tyrosinase. We verified that the extracts had the tyrosinase-inhibiting ability and melanin content effectiveness in mouse melanoma cell, B16-F10. In Figure 3(a), the extracts had revealed superior obvious suppressions even at a moderate quantity concentration to both tyrosinase activity and melanin content. Additionally, Figure 3(b) shows that melanin contents and tyrosinase activities were highly correlated under the same dose-responsive manner upon* C. osmophloeum* Kanehira treatments. Both tyrosinase activity and melanin content decreased in a similar dose-dependent tendency, when we increased dosages of extracts, indicating that the inhibition of cellular tyrosinase activity might induce the epidermal melanin reduction. But interestingly, extracts at the concentration 100 *μ*g/mL, the cell viability remained 52% (Figure 2), and the tyrosinase activity was 18% lower than cell viability (Figure 3(a)). Melanin content did not show evident reduction. On the contrary, with highest concentration of* C. osmophloeum *Kanehira extracts at 50 *μ*g/mL, the melanin content was decreased for low cell viability. As the tendency of tyrosinase activity was lower than cell viability, the melanin content was a bit higher than cell viability.

### 3.3. Measurement of* C. osmophloeum* Kanehira Extracts on Mushroom Tyrosinase Activity

**Figure 4:**
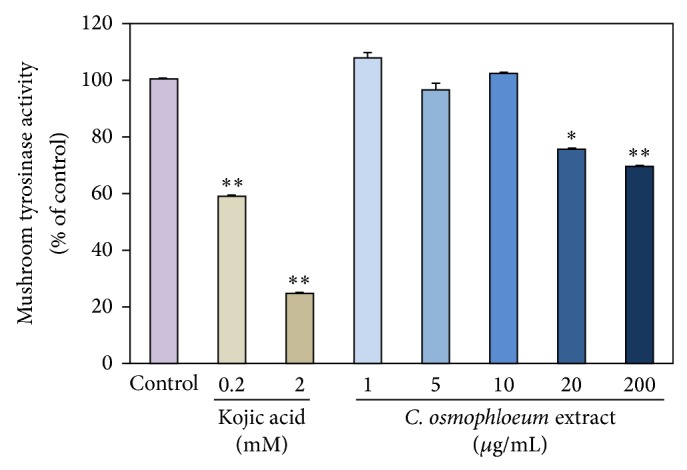
The inhibitory effects of various concentrations of* C. osmophloeum* extracts and kojic acid on mushroom tyrosinase for 5 minutes. Data: the mean value ± SD (triplicate values in three independent experiments); ∗ < 0.01, ∗∗ < 0.001.

We previously reported that UV exposure may induce the oxidative stress which is prone to be skin darkening and ROS generation for tumor progression [22, 23]. For the prevention of skin darkening and the hyperpigmentation, we evaluated the inhibitory effects of* C. osmophloeum* Kanehira extracts using* in vitro* mushroom tyrosinase inhibitory assay. The inhibition effectiveness of* C. osmophloeum* Kanehira extracts demonstrates moderate suppression to the activity of mushroom tyrosinase at 200 *μ*M (Figure 4). Accordingly, these compounds have the potential use for supplements in industry of cosmetics and pharmaceuticals.

### 3.4. Evaluation of the* In Vivo* Wound Size Assay

**Figure 5:**
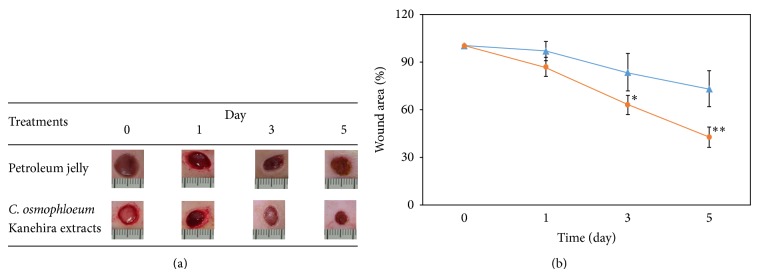
The wound healing of* C. osmophloeum* extracts on animal model. (a) Wound healing after 0, 1, 3, and 5 days after injury. After the full thickness excisions of 1 cm in diameter were made, the petroleum jelly (8% DMSO, w/v) or 0.8 mg* C. osmophloeum* extracts covered on the hurt. For injury group, wounds were not covered for control. At the first day, the wound healing area from petroleum jelly (8% DMSO) group was smaller than that of* C. osmophloeum *group until 5 days. (b) The differences between wounds of petroleum jelly (8% DMSO, w/v, blue solid triangle) and 0.8 mg* C. osmophloeum* group (orange solid circle) were statistically significant at day 5. Data: the mean value ± SD (triplicate values in two independent experiments); ∗ < 0.01, ∗∗ < 0.001.

In order for* C. osmophloeum *Kanehira extracts to be utilized as a dermal agent, they should exhibit minimal toxicity towards normal tissues. The wound-healing performance of the* C. osmophloeum* Kanehira extracts was measured by an animal model in terms of the full thickness wound assay and monitored by image analysis of excision wound area. We discovered that the mice displayed no obvious evidences of gross toxicity during the course of the treatment period. The body weight of the mice was also monitored at an interval of every other day over the course of this study. The results showed that the body weight of mice in the treatment and the control groups was not significantly different over the duration of the experiment (data not shown). Additionally, the mean values of the heart, liver, and kidney weights after sacrifice between these two groups of mice were not significantly changed. The wound areas of both petroleum jelly (8% DMSO, w/v) and 0.8 mg extract treatment groups were decreased in a time-dependent manner (Figure 5). Wound areas of extract-treatment groups at days 0, 1, 3, and 5 were 100 ± 0.1%, 86.7 ± 5.8%, 63.0 ± 6.1%, and 42.7 ± 6.4%, respectively, which were smaller than those of petroleum jelly group (100 ± 0.4%, 96.7 ± 5.8%, 83.3 ± 11.5%, and 73.0 ± 11.3%) (Figure 5(a)). In the beginning of the wound creation, the experimental group displayed smaller area than that of petroleum jelly group which showed a constant repair trend after 3 days. The* C. osmophloeum* Kanehira-treated wound healing displayed over 30% and 50% wound area closure after 3 and 5 days, respectively (Figure 5(b)). Therefore, the repairing ability and the wound shrinking ratio of the extract experimental group were higher and more effective.

### 3.5. Antioxidative Properties of* C. osmophloeum* Kanehira Extracts

Accumulating evidence shows that free radical increases can lead to skin melanin overexpression and speed up the variety of oxidations of lipids in manufactured food and cosmetics [1]. Natural antioxidants, particularly free radical eliminating abilities, are essential to skin caring. In Table 1, the inhibitory data for extracts to DPPH from 0 to 250 *μ*g/mL were found. Based on this information, extracts showed a dose-dependent manner in our testing conditions and might function as chain-breaking agents or radical ion neutralizers inhibiting the generation of excess free radicals in reference to control of 100 *μ*M vitamin C.

Several literatures reported that reducing power and metal ions-chelating ability from a reagent are commonly displayed through antioxidative effect [18, 19]. In Table 1, the ferrous ion chelating data of* C. osmophloeum* Kanehira extracts were found. When the complex formation of ferrozine and Fe(II) is interfered, it was detectable spectrophotometrically. Therefore,* C. osmophloeum* Kanehira extracts were demonstrated to have few Fe(II) chelating belongings.

The measurement of reducing power is one of the easy and effective methods by reacting with a Fe(III) ferricyanide complex [18, 19]. The solution color changes from light yellow to dark green and blue depended on the level of antioxidants within samples. As shown in Table 1,* C. osmophloeum *Kanehira extracts reducing powers were compared to similar antioxidative power with 3-*tert*-butyl-4-hydroxyanisole (BHA) at 100 *μ*M.

## 4. Conclusion

Various biochemical characterization properties of* C. osmophloeum *Kanehira extracts were explored within this study. In the cellular experiments, extracts had tyrosinase inhibition effect and melanin content diminution but were cytotoxic. The extracts might have lower cell viabilities to contribute to the results in both inhibited tyrosinase activity and reduced melanin content. In the animal model, we found that the extracts could repair the wound. The antioxidative assays suggested that high concentration of extracts had moderate antioxidative abilities in terms of DPPH scavenging capacity and reducing power ability except metal chelating. In the future, we will analyze purification from* C. osmophloeum *Kanehira extracts to find an effective antioxidant and tyrosinase inhibitor.

## Figures and Tables

**Table 1:** Measurement of *C. osmophloeum* extracts on antioxidant experiments. The data were expressed as a mean value in three independent experiments.

*C. osmophloeum* extract (*μ*g/mL)	DPPH scavenging (%)	Metal chelating ability (%)	Reducing power (OD 700)
1	≤10.0	≤10.0	0.09 ± 0.00
10	≤10.0	≤10.0	0.10 ± 0.00
50	≤10.01	≤10.0	0.18 ± 0.02
100	13.23 ± 0.01	≤10.0	0.27 ± 0.03
250	38.97 ± 0.02	≤10.0	0.48 ± 0.02

Vitamin C^a^	80.82 ± 0.00	—	—
EDTA^b^	—	80.76 ± 0.01	—
BHA^c^	—	—	0.56 ± 0.03

“—” is no testing.

^a^Vitamin C was used as a positive control on DPPH assay at 100 *μ*M.

^b^EDTA was used as a positive control on metal chelating ability at 100 *μ*M.

^c^BHA was used as a positive control on reducing power at 100 *μ*M.
